# Risk predictors of infection and mortality in population of cardiac surgery patients on ecmo therapy

**DOI:** 10.1186/2197-425X-3-S1-A368

**Published:** 2015-10-01

**Authors:** T Bachleda, M Prusák, M Plimon, R Ristl, MJ Hiesmayr

**Affiliations:** Clinic of Anaesthesiology, ICM and Pain Therapy / Cardiac-Thoracic-Vascular Division, Medical University of Vienna, Vienna, Austria; Center for Medical Statistics, Informatics and Intelligent Systems, Medical University of Vienna, Vienna, Austria

## Introduction

The main hazardous events in the population of cardiac surgery patients on veno-arterial extracorporeal membrane oxygenation therapy (va-ECMO) are occurrence of infection and death.

## Objectives

To identify potential risk factors for either event and to study if the risk of death is changed after an infection has occurred.

## Methods

Retrospective trial involving 149 adult cardiac surgery patients undergoing va-ECMO therapy between 2008 and 2012. The variables considered as possible risk factors at baseline were sex, age, BMI, immune suppression therapy (IST), SAPS II score, circumstances of ECMO implantation (OR, not OR) and SIRS.

For the effect of baseline variables for time to death and on the risk of infection simple Cox models were calculated. A multiple regression model was calculated including all predictors found significant in the simple models. Hazard ratio (HR) and 95% confidence interval (CI) for infected and not infected were calculated in a simple Cox regression model with all patients and model excluding patients without infection before ECMO.

## Results

15 patients were removed from analysis due to incomplete data sets. We analyzed 134 patients involving 4.977 ICU days (mean 36,9) and 880 ECMO-days (mean 6,5). Eight patients had an infection before ECMO implantation.

The estimated HR of infection status for risk of death was close to 1, for both sets of patients taken into consideration: all patients (HR 0,927; CI 0,568-1,512; p = 0,76), patients without infection before ECMO (HR 1,036; CI 0,612-1,755; p = 0,895).

In the set of baseline risk factors on risk of death only age was found to be significant with an estimated HR of 1,039 per year (CI 1,016-1,061; p = 0,001). The estimated HR of infection status remains almost unchanged when adjusting for age at baseline: infection status (HR 1,082; CI 0,657-1,782; p = 0,756), age (HR 1,041; CI 1,018-1,065; p < 0,001).

Effect of IST on risk of death in a multiple Cox model also accounting for age was similar to the results from the simple model: age (HR 1,035; CI 1,013-1,058; p = 0,001), IST (HR 1,544; CI 0,837-2,846; p = 0,164).

In the set of baseline risk factors on risk of infection was significant absence of SIRS (HR 0,292; CI 0,132-0,648; p = 0,002), SAPS II score (HR 1,021; CI 1,007-1,036; p = 0,004) and IST (HR 0,414; CI 0,233-0,733; p = 0,003). This effect on risk of infection in multiple Cox model was similar: absence of SIRS (HR 0,335; CI 0,15-0,747; p = 0,008), SAPS II score (HR 1,021; CI 1,006-1,035; p = 0,004) and IST (HR 0,443; CI 0,249-0,789; p = 0,006).

## Conclusions

Age at admission seems to be a strong predictor of mortality in a mixed population of ECMO patients after cardiac surgery. Reliable predictors on risk of infection seem to be absence of SIRS, SAPS II score and immune suppression therapy. Risk of death does not increase with occurrence of an infection.

